# Comparison of Minced Cartilage Implantation with Autologous Chondrocyte Transplantation in an In Vitro Inflammation Model

**DOI:** 10.3390/cells13060546

**Published:** 2024-03-20

**Authors:** Robert Ossendorff, Lisa Grede, Sebastian Scheidt, Andreas C. Strauss, Christof Burger, Dieter C. Wirtz, Gian M. Salzmann, Frank A. Schildberg

**Affiliations:** 1 Department of Orthopedics and Trauma Surgery, University Hospital Bonn, 53127 Bonn, Germany; 2 Gelenkzentrum Rhein-Main, 65239 Hochheim, Germany; 3 Schulthess Clinic, 8008 Zurich, Switzerland

**Keywords:** autologous chondrocyte transplantation, minced cartilage implantation, inflammation model, tumor necrosis factor α, musculoskeletal immunology

## Abstract

The current gold standard to treat large cartilage defects is autologous chondrocyte transplantation (ACT). As a new surgical method of cartilage regeneration, minced cartilage implantation (MCI) is increasingly coming into focus. The aim of this study is to investigate the influence of chondrogenesis between isolated and cultured chondrocytes compared to cartilage chips in a standardized inflammation model with the proinflammatory cytokine TNFα. Articular chondrocytes from bovine cartilage were cultured according to the ACT method to passage 3 and transferred to spheroid culture. At the same time, cartilage was fragmented (<1 mm^3^) to produce cartilage chips. TNFα (20 ng/mL) was supplemented to simulate an inflammatory process. TNFα had a stronger influence on the passaged chondrocytes compared to the non-passaged ones, affecting gene expression profiles differently between isolated chondrocytes and cartilage chips. MCI showed less susceptibility to TNFα, with reduced IL-6 release and less impact on inflammation markers. Biochemical and histological analyses supported these findings, showing a greater negative influence of TNFα on the passaged pellet cultures compared to the unpassaged cells and MCI constructs. This study demonstrated the negative influence of TNFα on chondrogenesis in a chondrocyte spheroid culture and cartilage fragment model. Passaged chondrocytes are more sensitive to cytokine influences compared to non-passaged cells and chondrons. This suggests that MCI may have superior regeneration potential in osteoarthritic conditions compared to ACT. Further investigations are necessary for the translation of these findings into clinical practice.

## 1. Introduction

Cartilage injuries are detected with increased frequency [1]. Articular cartilage has a limited capacity for self-repair. When untreated, the risk of developing osteoarthritis is highly increased [2]. Therefore, various cartilage regenerative techniques have been developed to treat symptomatic cartilage defects [3]. Autologous chondrocyte transplantation (ACT) is the gold standard for the repair of large “traumatic” chondral defects (>2 cm^2^). As a two-step surgical approach, cartilage is harvested from non-weight-bearing regions and chondrocytes are isolated and expanded ex vivo. Finally, cells are seeded on a scaffold (mACT) or transferred to a spheroid culture (spheroid technique) [4,5]. In a second surgery, the transplant is implanted into the defect. ACT shows good clinical outcomes (>10 years) and higher regenerate quality compared to alternative procedures, such as microfracture [6,7,8]. Nevertheless, the complication rate increases over time, with a revision rate of up to 30% in follow-up 8–11 years after surgery [8]. As a new technique, minced cartilage implantation (MCI) has come into focus. MCI is performed autologously as a single surgical procedure. Cartilage is harvested from the edge of the defect, minced into fragments (<1 mm^3^) and re-implanted with autologous platelet-rich plasma, fibrin glue or membranes [9]. The mincing of cartilage activates chondrocytes to migrate, proliferate and differentiate, which results in a hyaline-like neo-tissue [9]. Furthermore, a major advantage of the MCI procedure is the intact functional unit of the chondrocyte and pericellular matrix, which is called the “chondron”. This pericellular matrix plays an important role in preserving joint homeostasis [10,11]. The initial clinical studies show a promising outcome of MCI at up to 2 years of follow-up [12,13,14] with limited evidence. Cartilage repair is performed in a majority (60%) of degenerative, non-traumatic defects (data from the German Society for Orthopaedics and Trauma cartilage registry) [15]. This disturbed joint homeostasis with increased inflammation and catabolic processes contributes to a higher risk of treatment failure [16]. The proinflammatory cytokine TNFα is well-known as a central factor of osteoarthritic progression [2]. Persistent elevated levels of this cytokine lead to disturbed joint homeostasis with increased inflammation and stimulation of catabolic processes [17]. The quality of cartilage regenerate is affected by biomechanical changes in the transplant leading to increased stiffness and impaired contractile function [18]. To the best of our knowledge, there is a lack of evidence on which cartilage repair technique results in the best cartilage regenerate quality in osteoarthritic surroundings.

The aim of this study was to compare the influence of inflammation on transplant maturation between MCI and ACT (spheroid technology) by supplementation of the proinflammatory cytokine TNFα. Secondly, we aimed to analyze the effect of in vitro cell expansion as a central part of the ACT procedure through the comparison of primary non-passaged chondrocytes (P0) to passaged chondrocytes (P3).

## 2. Materials and Methods

### 2.1. Experimental Design

**Figure 1:**
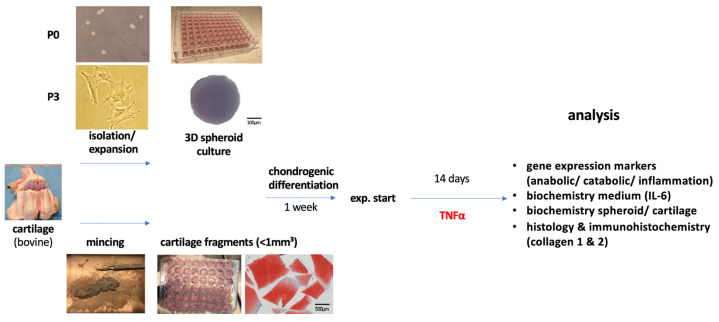
Schematical overview of the experimental design. Bovine chondrocytes were extracted from bovine fetlock joints, remained at passage 0 or expanded to passage 3 and transferred in 3D spheroid culture for chondrogenic differentiation (spheroid diameter 500 µm). Microscopy shows the typical round-cell morphology of the chondrocyte (P0, non-passaged) and fibroblast shape of the P3 chondrocytes. The MCI group was minced into pieces of <1 mm^3^. Cytokine supplementation with TNFα was performed over a period of 14 days. Analyses included RT-PCR, biochemical and histological evaluation.

Articular cartilage was harvested from bovine fetlock joints (age 6–24 months); the animals were euthanized by a local butcher for food production without the need for ethical approval (Figure 1). The articular cartilage was fragmented using a scalpel (<1 mm^3^) according to the minced cartilage procedure. At the same time, articular chondrocytes were isolated.

For passage 0, no cell expansion was performed, and chondrocytes were directly transferred into pellet culture. In a third group, chondrocytes were expanded to passage 3 (5–8 population doublings). All samples underwent 1 week of culture. Subsequently, supplementation of the proinflammatory cytokine TNFα (20 ng/mL) was performed to simulate inflammation. The start of cytokine stimulation was defined as day 0 and followed by a medium change three times per week. The experiment included four different independent donors with technical triplicates for each analysis and was terminated after two weeks.

### 2.2. Isolation and Culture of Minced Cartilage Samples

According to the minced cartilage procedure, the articular cartilage was minced using a scalpel (blade No. 10) into pieces of <1 mm^3^ in size [19]. The cartilage was pre-wetted with phosphate-buffered saline solution (PBS) (Thermo Fisher Scientific, Waltham, MA, USA) to avoid the tissue drying and losing weight. The cartilage pieces were equally distributed in a 48-well plate (8 fragments per well) and suspended in 17 mg/mL fibrinogen solution and 0.5 U/mL thrombin solution (Sigma-Aldrich, St. Louis, MO, USA) for 3-dimensional clot formation. The samples were cultured in a chondropermissive medium (1 mL/well) without growth factors, including DMEM, with 10% FBS, 60 µg/mL ascorbic acid phosphate (Sigma-Aldrich), 40 µg/mL L-proline (Sigma-Aldrich), 1% non-essential amino acids (Sigma-Aldrich), 1% ε-aminocaproic acid (Sigma-Aldrich) and 0.2% Primocin (InViVoGen, San Diego, CA, USA).

### 2.3. Isolation and Culture of Articular Chondrocytes

The cartilage fragments (<10 mm^3^) were first digested with pronase for 90 min (0.1%; Merck, Darmstadt, Germany), followed by digestion in 600 units/mL collagenase 2 (Worthington, Lakewood, NJ, USA) for 14 h. For the passage 0 group, chondrocytes were counted, transferred to 96 v-bottom non-adherent well plates (Greiner, Kremsmünster, Austria) and centrifuged at 500× *g* 10 min for pellet formation (250 × 10^3^ cells per well). Spheroids were cultured 1 week in free swelling conditions in a chondropermissive culture medium without the addition of exogenous growth factors, including DMEM, with 10% FBS, 60 µg/mL ascorbic acid phosphate (Sigma-Aldrich, St. Louis, MO, USA), 40 µg/mL L-proline (Sigma-Aldrich), 1% nonessential amino acids (Gibco), 1% ε-aminocaproic acid (Sigma-Aldrich) and 1% Pen/Strep (Sigma-Aldrich).

For the passage 3 group, according to ACT procedure the extracted chondrocytes were seeded at a density of 16.7 × 10^3^ cells/cm^2^ in Dulbecco’s modified Eagle medium (high glucose) (Thermo Fisher Scientific, Waltham, MA, USA) and 10% fetal bovine serum (FBS) (Bio & Sell, Feucht, Germany). Cells were passaged when 90% confluency was reached. Pre-digestion was performed in 300 units/mL collagenase 2 (30 min) for cleavage of trypsin-resistant extracellular components (e.g., collagens), followed by digestion with 0.05% trypsin-EDTA (Thermo Fisher Scientific, Waltham, MA, USA) (5–20 min, depending on the passage with longer detaching time in early passages). Cells were seeded at the same density. Medium change was repeated every second day with growth medium including DMEM + 10% FBS. The cell expansion time from P0 to P3 was, on average, 2 weeks. The cells were transferred at passage 3 in a spheroid culture as previously described and cultivated for 1 week in free swelling conditions in a chondropermissive culture medium.

### 2.4. Chondrocyte Inflammation Model

TNFα (R&D Systems, Minneapolis, MN, USA) was supplemented to induce inflammation with a concentration of 20 ng/mL in a standardized model for better comparison with previous studies [17,20]. TNFα was added to the medium six times over 14 days with every medium change, and the medium was collected for further biochemical analysis.

### 2.5. Biochemical Analysis

All samples were digested for biochemical analysis in a proteinase K solution (0.5 mg/mL; Roche, Basel, Switzerland) for 14 h. DNA was quantified by spectrofluorimetry with Hoechst dye solution 33,528 (Applied Biosystems, Waltham, MA, USA) against the standard calf thymus DNA (Sigma-Aldrich, St. Louis, MO, USA), as previously described [21]. Chondrocyte spheroids were analyzed for glycosaminoglycan (GAG) content by a 1,9-dimethyl-methylene blue (DMMB; Sigma-Aldrich) dye binding assay. The standard was bovine chondroitin sulfate (Sigma-Aldrich) [22]. The medium was analyzed for all the separate time points for its IL-6 concentration using a bovine IL-6 ELISA assay kit (Kingfisher Biotech, St Paul, MN, USA).

### 2.6. RNA Extraction, Reverse Transcription and Gene Expression Analysis

A tissue-lyzer system (Qiagen, Hilden, Germany) was used for homogenization in 1 mL TRI reagent (Molecular Research Center, Cincinnati, OH, USA) for 10 min at 30 Hz. RNA was extracted by a standardized precipitation method with bromochloropropane (BCP, Sigma-Aldrich) in a volume ratio of 1:10 for phase separation, and RNA clean-up with a tissue-specific column-based extraction kit (Qiagen). For cDNA synthesis, a reverse transcription was performed with TaqMan reverse transcription reagents (Applied Biosciences) in 1 µg total RNA. The Taq-Man Real-Time PCR system (Applied Biosystems) analyzed gene expression in the TaqMan master mix and custom-made bovine primer and probes (Applied Biosystems) for the specific anabolic markers collagen 2, aggrecan and COMP (cartilage oligomeric protein), the dedifferentiation marker collagen 1, the catabolic markers MMP-3, MMP-13 (matrix metalloproteinases 3 and 13) and the inflammation markers IL-6 and IL-8 (interleukin-6 and −8) were used. The primers and TaqMan probes (Applied Biosystems) were designed from GenBank and have been previously described [23,24]. The 18S ribosomal RNA was used as the housekeeping gene of eukaryotic cells, and gene expression was measured relative to the endogenous control. The threshold cycle (CT) values were normalized to the mean CT values of 18S (ΔCT) and normalized to day 0 (ΔΔCT). The relative mRNA expression was calculated with the 2^−ΔΔCT^ method and normalized by the natural logarithm to avoid skew deviations.

### 2.7. Histology and Immunohistochemistry

The spheroids and MCI samples were fixed in 70% methanol, followed by paraffin fixation with a carousel tissue processor for 24 h including incubation in 70% ethanol, 96% ethanol, 100% ethanol, xylene and paraffin. The paraffin-embedded samples were cut by a microtome into 5 µm sections. Safranin O and Fast Green (Sigma-Aldrich) staining was performed to evaluate cell morphology and extracellular matrix deposition. The paraffin sections were stained with Weigert´s Haematoxylin (Sigma-Aldrich) for 10 min, followed by 0.02% Fast Green (Sigma-Alrich) in ultrapure (ddH_2_O) water for 6 min and 0.1% Safranin O for 10 min. Collagen type 1 and collagen type 2 immunohistochemistry was performed with the Vectastain avidin-biotin-complex based (Vector Laboratories, Newark, CA, USA) staining method, as previously described, with antibodies against collagen 1 (Arcis, Worrington, United Kingdom) or collagen 2 (DSHB, Iowa, IA, USA) and PBS as the negative control, followed by a biotinylated IgG antibody, DAB (3-3´-diaminobenzidine; Vector Laboratories). Mayer hematoxylin (Sigma-Aldrich) was used to counterstain the sections [20]. A semiquantitative analysis was performed for the Safranin O Fast Green stain and the IHC of collagen 1 and 2 by pixel analysis with evaluation of the percentage of stain against the background stain.

### 2.8. Statistics

Statistical analysis was performed with SPSS (v 24; IBM, Armonk, NY, USA). Normal distribution was tested with the Kolmogorov–Smirnov test. All results did not show a normal distribution. Therefore, the non-parametric Wilcoxon–Mann–Whitney test was applied to test for significant differences among the defined groups as independent variables and analytical results as dependent groups. Statistical significance was defined at *p* < 0.05. Graphpad Prism 9 (Graphpad Software Inc., San Diego, CA, USA) was used for graphical design.

## 3. Results

### 3.1. Chondrogenic Differentiation Potential Is Not Affected in MCI Culture

**Figure 2:**
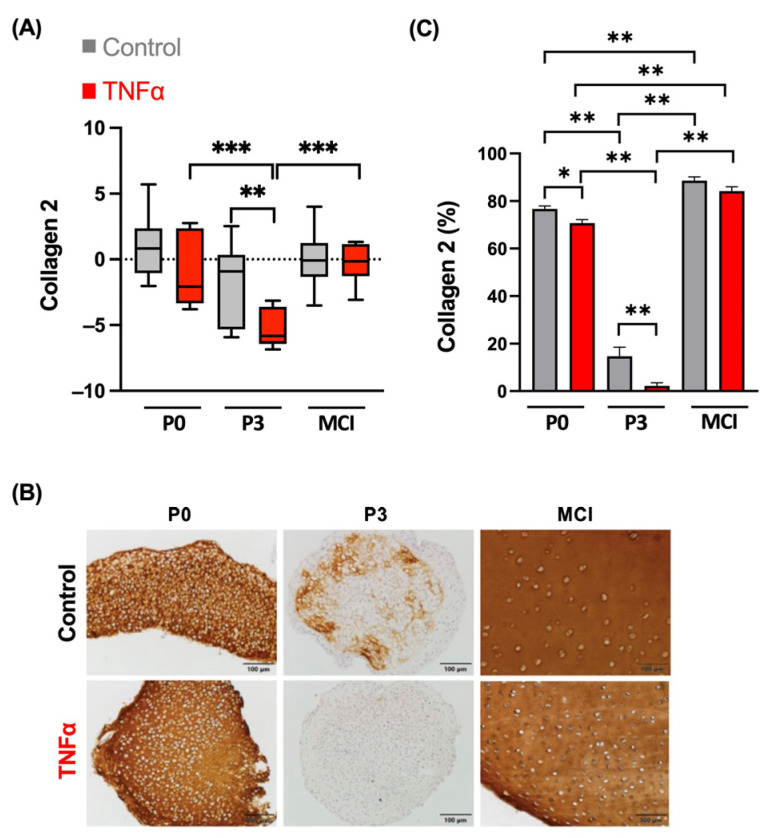
(**A**) Gene expression analysis of collagen 2 of spheroids (P0 and P3) and cartilage fragments (MCI) in an inflammation model with TNFα. Results from four independent donors and three technical replicates are normalized to 18S as internal control, transformed by natural logarithm and visualized in box plots. (**B**) Immunohistochemical staining of chondrocyte spheroid constructs and cartilage fragments for collagen 2. Negative controls are shown in Supplementary Figure S1A. (**C**) Semi-quantitative analysis of stain intensity of collagen 2 compared to background stain hematoxylin and eosin. * *p* < 0.05, ** *p* < 0.01, *** *p* < 0.001. P0, non-passaged chondrocytes; P3, chondrocyte passage 3; MCI, minced cartilage; TNFα, tumor necrosis factor α.

The gene expression level of the chondrogenic differentiation marker collagen 2 was significantly reduced by supplementation of TNFα in the P3 spheroid constructs compared to the control (*p* = 0.003, Figure 2A). This effect was confirmed at the protein level by immunohistochemical analysis. Collagen 2 synthesis in chondrocytes was strongly reduced by TNFα (Figure 2B,C). The P0 and MCI samples did not indicate any influence of TNFα on collagen 2 expression. A slight decrease in collagen 2 retention caused by cytokine treatment could be detected for P0 in the semiquantitative analysis (*p* = 0.038). Gene expression did not show significant differences between P3 and the other groups.

**Figure 3:**
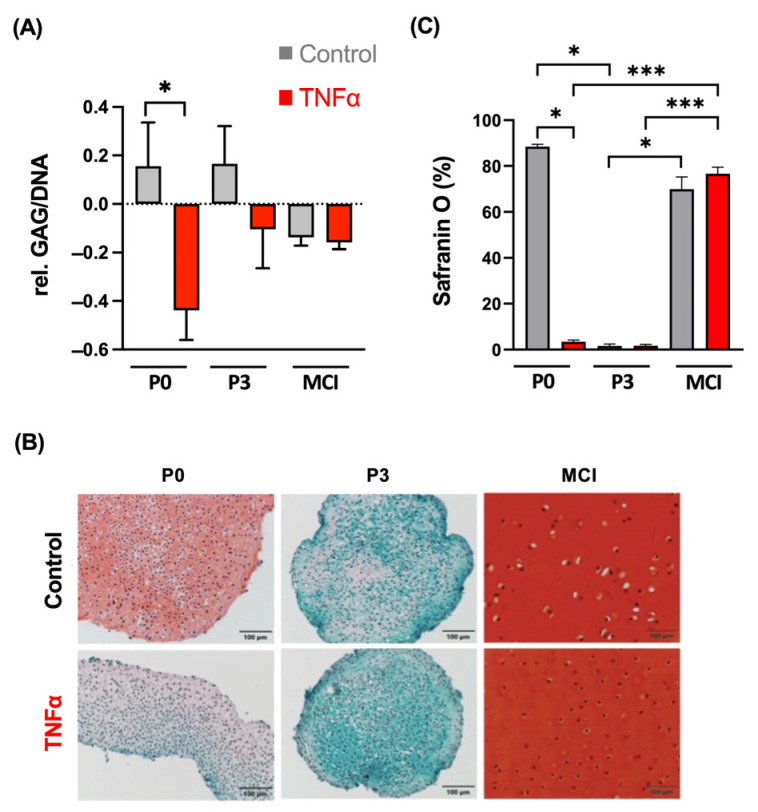
(**A**) Biochemical quantification of GAG samples relative to DNA (µg/µg) and day 0 from four independent donors and three technical replicates. (**B**) Safranin O Fast Green staining of chondrocyte spheroid constructs and cartilage fragments in an inflammation model with TNFα. (**C**) Semi-quantitative analysis of stain intensity of Safranin O compared to background stain Fast Green. * *p* < 0.05, *** *p* < 0.001. P0, non-passaged chondrocytes; P3, chondrocyte passage 3; MCI, minced cartilage; TNFα, tumor necrosis factor α.

The relative synthesis of glycosaminoglycans (GAGs) was significantly reduced by the supplementation of TNFα in P0 spheroids over 14 days of culture (*p* = 0.029; Figure 3). In the qualitative analysis, the Safranin O Fast Green staining demonstrated a high proteoglycan loss in the TNFα-treated P0 constructs compared to the control. The P3 spheroids showed a low content of proteoglycans, indicating dedifferentiation without significant differences between cytokine stimulation and the control. The MCI constructs showed a characteristic structure of hyaline cartilage with an extracellular matrix with high amounts of proteoglycans, as seen by the intense red staining of the histological section. The MCI samples did not show any effects by the supplementation of TNFα.

**Figure 4:**
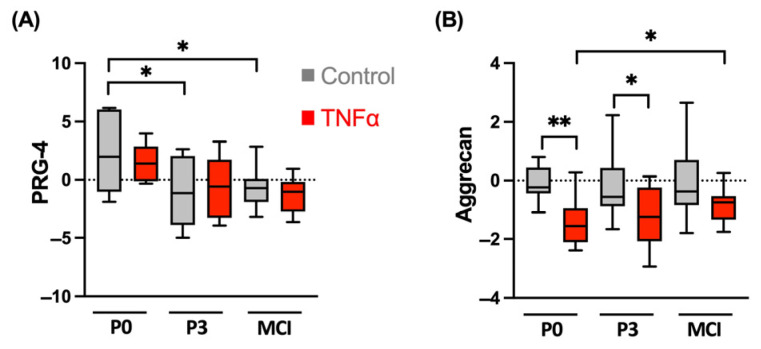
Gene expression analysis of the proteoglycans (**A**) proteoglycan-4 (PRG-4) and (**B**) aggrecan in spheroids and cartilage fragments in an inflammation model with TNFα from four independent donors and three technical replicates. Results are normalized to 18S as internal control, transformed by natural logarithm and visualized in box plots. * *p* < 0.05, ** *p* < 0.01.

On the gene expression level, the anabolic marker PRG-4 was significantly higher in the P0 control spheroids compared to in the P3 (*p* = 0.037; Figure 4) and MCI (*p* = 0.017) samples. There was no specific effect of the proinflammatory cytokine TNFα. The expression of the proteoglycan aggrecan was significantly reduced by supplementation of TNFα in the P0 spheroids (*p* = 0.002) and P3 constructs (*p* = 0.026) compared to the control. Cytokine treatment did not affect the gene expression of aggrecan in the MCI samples.

### 3.2. Collagen 1 Synthesis Is Not Influenced by TNFα in MCI Samples

**Figure 5:**
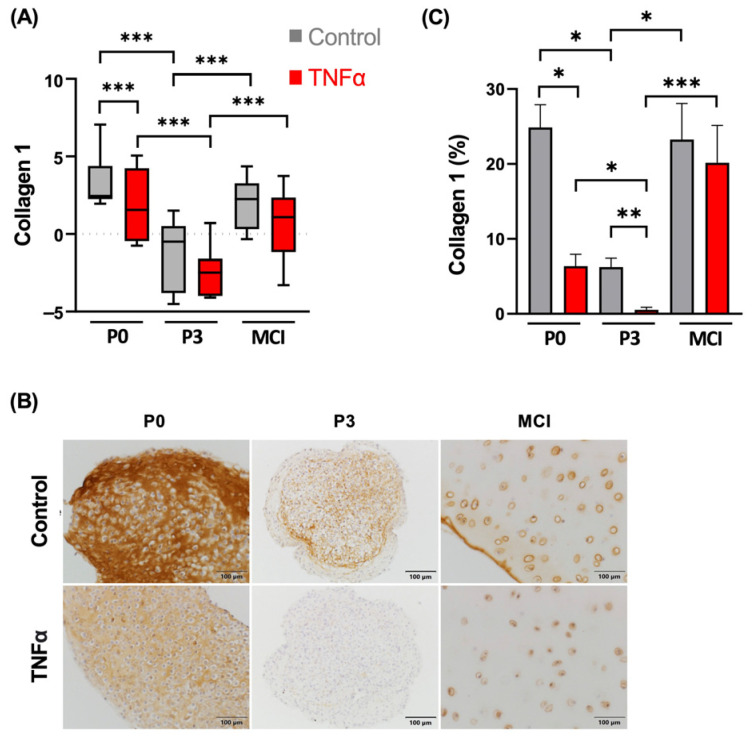
(**A**) Gene expression analysis for collagen 1 of chondrocyte spheroids and cartilage fragments in an inflammation model with TNFα from four independent donors and three technical replicates. Results are normalized to 18S as internal control, transformed by natural logarithm and visualized in box plots. (**B**) Immunohistochemical staining of chondrocyte spheroid constructs and cartilage fragments for collagen 1. Negative controls are shown in Supplementary Figure S1B. (**C**) Semi-quantitative analysis of stain intensity of collagen 1 compared to background stain hematoxylin and eosin. * *p* < 0.05, ** *p* < 0.01, *** *p* < 0.001. P0, non-passaged chondrocytes; P3, chondrocyte passage 3; MCI, minced cartilage; TNFα, tumor necrosis factor α.

The gene expression level of collagen 1 was significantly higher in the P0 control samples compared to the samples treated with TNFα (*p* < 0.001; Figure 5), which resulted in lower synthesis at the protein level, which is evident by immunohistochemistry. The gene expression of collagen 1 was significantly lower in the P3 chondrocytes compared to the P0 chondrocytes in the presence and absence of TNFα (*p* < 0.001). The collagen 1 content in IHC was lower in the P3 and MCI constructs compared to day 0. At the gene expression level, differences between the control and cytokine stimulation did not reach significance for these groups at day 14, which can be a reason for the late time point. Reduced staining of collagen 1 due to the supplementation of TNFα was detected in the immunohistochemical assessment of the P3 spheroids, which was significant in the semi-quantitative analysis (*p* = 0.003; Figure 5C).

### 3.3. MMPs Show Different TNFα-Induced Patterns in ACT and MCI Transplants

**Figure 6:**
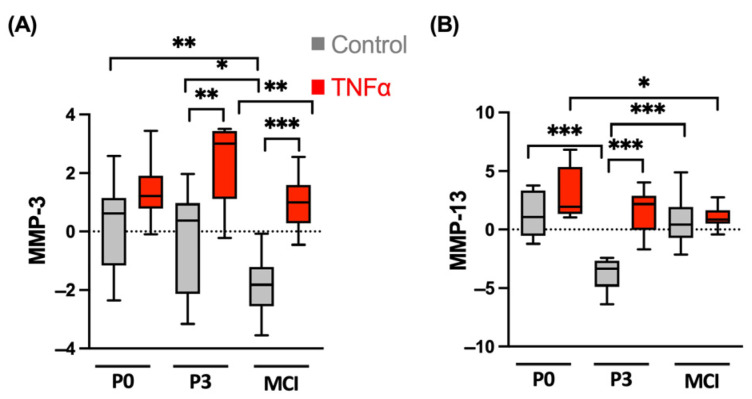
Gene expression analysis of (**A**) matrix metalloproteinases MMP-3 and (**B**) MMP-13 of chondrocyte spheroids and cartilage fragments in an inflammation model with TNFα from four independent donors and three technical replicates. Results are normalized to 18S as internal control, transformed by natural logarithm and visualized in box plots. * *p* < 0.05, ** *p* < 0.01, *** *p* < 0.001. P0, non-passaged chondrocytes; P3, chondrocyte passage 3; MCI, minced cartilage; TNFα, tumor necrosis factor α.

The gene expression of the catabolic markers MMP-3 and MMP-13, as important enzymes of extracellular matrix cleavage, were generally upregulated by the supplementation of TNFα. For MMP-3, gene expression was upregulated compared to the control in the P3 spheroids (*p* = 0.002; Figure 6) and the MCI constructs (*p* < 0.001).

The highest expression was detected in the P3 chondrocytes and was significantly higher compared to MCI (*p* = 0.031). The MMP-3 expression of the P0 spheroids did not result in significant differences between the control and the TNFα-supplemented groups. For MMP-13, the gene expression of the controls in the P3 chondrocyte spheroids was significantly lower compared to P0 (*p* = 0.001) and MCI (*p* < 0.001). Supplementation of the proinflammatory cytokine TNFα resulted in increased MMP-13 expression in the P3 chondrocyte spheroids compared to the control (*p* < 0.001), but not in the P0 and MCI samples.

### 3.4. ACT Constructs Are More Sensitive to TNFα-Induced Inflammation Compared to MCI Grafts

**Figure 7:**
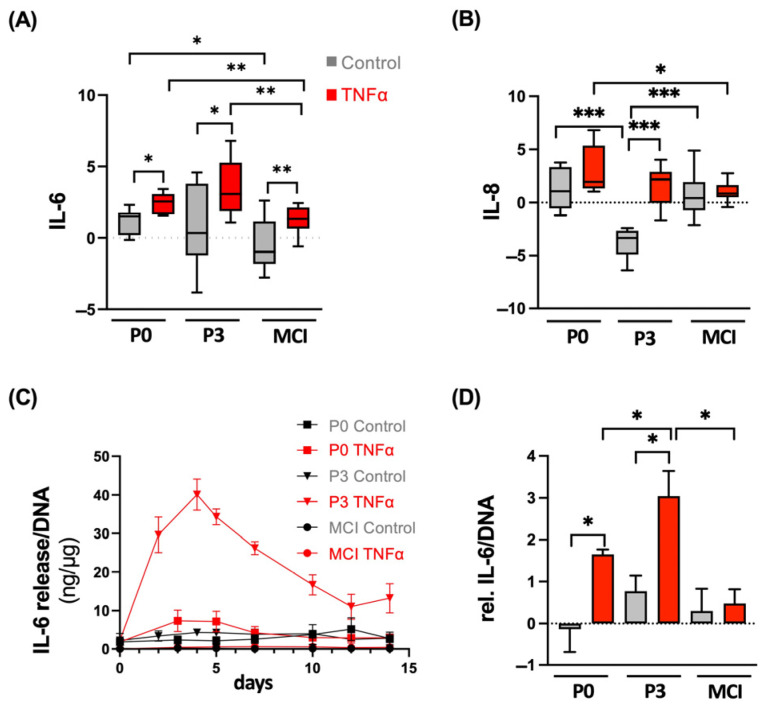
Gene expression analysis of (**A**) IL-6 and (**B**) IL-8. Results are normalized to 18S as internal control, transformed by natural logarithm and visualized in box plots. (**C**) Dynamic evaluation of IL-6 release (ng) at indicated time points (days). (**D**) Cumulative quantification of IL-6 per DNA (ng/µg) relative to day 0. * *p* < 0.05, ** *p* < 0.01, *** *p* < 0.001. P0, non-passaged chondrocytes; P3, chondrocyte passage 3; MCI, minced cartilage; TNFα, tumor necrosis factor α. Analysis was performed from four independent donors and three technical replicates.

The inflammatory response was evaluated by a gene expression analysis of the proinflammatory cytokines IL-6 and IL-8 and a quantitative protein analysis of IL-6. TNFα stimulation of the P3 chondrocyte spheroids and MCI constructs resulted in upregulation of IL-6 compared to the control (P3 TNFα vs. control, *p* = 0.022; MCI TNFα vs. control, *p* = 0.003; Figure 7A). The elevation was significantly higher in the chondrocytes of the P3 spheroids compared to the chondrocytes in the MCI samples (*p* = 0.002). Furthermore, the gene expression of IL-6 in the untreated control was lower in the MCI (*p* = 0.002) and P0 chondrocytes (*p* = 0.012) compared to the P3 spheroid culture. IL-8 expression was stimulated in chondrocytes of the P3 spheroid constructs in the presence of TNFα (*p* = 0.001). At the same time, gene expression of the P3 control chondrocytes was lower compared to that of the P0 (*p* = 0.001) and MCI (*p* < 0.001) constructs. The P0 and MCI constructs did not show any TNFα-related effect on the gene expression of IL-8. In terms of the protein level, IL-6 release into the medium showed a dynamic change over the 14 days, with peak levels at day 4, with the highest concentrations in the TNFα-stimulated P3 spheroids (>40 ng/mL). The peak concentrations were five times higher for the P3 spheroids than for the P0 spheroids. The cumulative analysis indicated the significantly enhanced release of IL-6 for the P0 (*p* = 0.01) and P3 spheroids (*p* < 0.001) by the supplementation of TNFα compared to the control. The P3 chondrocyte stimulation by TNFα was significantly higher compared to that of the P0 spheroids (*p* = 0.004) and MCI samples (*p* < 0.001).

## 4. Discussion

### 4.1. Key Findings

This study compared, for the first time, the differential effects on chondrocytes between the MCI and ACT spheroid techniques in an inflammation model with the proinflammatory cytokine TNFα over 14 days of culture. The TNFα stimulation of passaged chondrocytes according to the ACT procedure resulted in the upregulation of catabolic makers and downregulation of anabolic markers in gene expression analysis and the loss of proteoglycans and collagen 2 at the protein level. Inflammation was enhanced by upregulation of the cytokines IL-6 and IL-8. These effects were significantly lower in the MCI culture and spheroid culture of the P0 chondrocytes. Consequently, the ACT spheroid constructs were more sensitive to the TNFα stimulus compared to MCI, an effect that is enhanced by the cell expansion process. The results showed a high reproducibility between four independent donors.

### 4.2. MCI Counteracts Anti-Catabolic and Catabolic Effects of TNFα

Symptomatic cartilage defects are treated with cartilage repair methods according to defect size [25]. Data from the German Society of Orthopaedics and Trauma registry uncovered that ACT was often performed in osteoarthritic surroundings [15]. In these conditions, inflammation is stimulated by proinflammatory cytokines, such as TNFα. Persistent inflammation affects the transplant quality and increases the risk of treatment failure, pain and progression of osteoarthritis [16]. Previous studies demonstrated that TNFα is a suppressor of extracellular matrix synthesis, resulting in reduced proteoglycan and collagen production [20,26]. In our experiment, matrix synthesis was affected in P0 and P3 spheroids, with the strongest effects in the culture of expanded chondrocytes. The ECM synthesis of the MCI constructs was not influenced by TNFα. MMPs are catabolic markers indicating cartilage degradation and are typically increased in osteoarthritic surroundings [27]. In this study, MMP-3 and MMP-13 were stimulated by TNFα. MMP-3 activity was increased in the P3 spheroids and MCI culture, but not in the P0 spheroids. Nevertheless, MMP-3 activity was also reported as a sign of ectopic cartilage formation [28]. For MMP-13, there was only a stimulative effect in the P3 spheroid culture.

### 4.3. Pericellular Matrix as Key of Chondrogenic Potential

The extracellular matrix contains the pericellular matrix (PCM), territorial and interterritorial zone. Chondrocytes are embedded in the pericellular matrix, which contains a complex network of proteoglycans, collagen 6, other collagens and non-collagenous proteins. The functional unit of the PCM and the chondrocytes is called the chondron. The PCM plays a major role in metabolic activity and homeostasis [29]. The advantage of MCI is the minimal manipulation of cartilage without the need for an ex vivo cell culture. The chondral structure is therefore not affected by the mincing procedure. Previous studies demonstrated that the PCM modulates anabolic and catabolic processes. Vonk et al. compared goat chondrons with isolated chondrocytes in an in vitro culture over 25 days in alginate beads [11]. Preservation of the PCM resulted in higher collagen 2 expression, cross-linked collagen 2 in Western blot analysis and an increased amount of proteoglycans. Hing et al. reported that the PCM provides a barrier when exposed to changes in osmolarity [30]. This integrity of the chondral structure may also contribute to the lower TNFα influence on the MCI samples that was seen in our study. As a result, the inflammatory stimulus was counteracted with equal IL-6 levels at the protein level and no upregulation of IL-8 expression in the MCI constructs compared to the control.

### 4.4. Mincing Procedure as Factor of Chondrocyte Activation

The central mechanism of MCI is the activation of chondrocytes by cutting the cartilage into small pieces. Albrecht et al. first described an induction of cartilage regeneration in a rabbit model by proliferation and differentiation of cartilage fragments [31]. With a sharp instrument (scalpel, mincing device) the vitality of the chondrocytes is not affected [32,33]. For biological activity, it is important to cut the cartilage into the smallest size (<1 mm^3^) to form a paste-like appearance [9,34]. In our study, the induced biological activation of the chondrocytes was not different between the control group and the group that underwent TNFα stimulation, which is a sign of counteraction of the cytokine stimulus. The underlying mechanisms of chondrocyte activation have not been clearly analyzed previously. This would be essential to prove the advantage of MCI over other approaches.

In the MCI technique, cartilage fragments are embedded in autologous or allogenic fibrin glue for better adhesion and tissue integration [35]. In autologous fibrin sealants, chondrocytes show a higher proliferation, migration and differentiation potential due to included growth factors. In our model, there was no significant effect on chondrocyte differentiation and ECM production by the use of fibrin sealant.

### 4.5. Cell Expansion Process in ACT Increases the Sensitivity to Inflammatory Stimuli

In autologous chondrocyte implantation, a two-step surgical approach with a cost-extensive culture process is necessary. In this procedure, chondrocytes are expanded up to passage 3 to expand the number of cells. Nevertheless, cell culture results in a rapid dedifferentiation of the chondrocytes. Wang et al. [36] compared P0 and P3 chondrocytes in an in vitro culture model with a knee-specific bioreactor. Primary chondrocytes demonstrated the strongest chondrogenic gene expression profile and the most intense extracellular matrix deposition. This is in line with our findings.

### 4.6. Limitations and Outlook

This study focused on the comparison of MCI and ACT transplants (P3), and an unpassaged spheroid culture (P0), in an inflammation model over 14 days of culture. A longer culture was not performed, which is a limitation of the in vivo situation. Furthermore, there was no mechanical stimulation, which would be the normal situation in in vivo surroundings. For higher standardization, a bovine model was selected, which shows a high reproducibility of the results between the different donors. For clinical translation, the described effects need to be confirmed in humans. For ACT approaches, it is necessary to isolate chondrocytes for expansion before re-implanting them into the lesion site with a well-defined minimal cell density. In contrast to that, it is unclear how many chondrons are necessary to sufficiently treat chondral lesions. What about osteochondral lesions, i.e., lesions that reach the bone? How do these chondrons react to biological signals from this area? Does this limit the application of the MCI approach to certain types of lesions? Further preclinical and clinical studies are necessary to compare the influence of inflammation on cartilage transplants of different cartilage repair methods, including Minced Cartilage Implantation and Autologous Chondrocyte Implantation.

## 5. Conclusions

This study demonstrated that passaged chondrocytes according to ACT are more sensitive to cytokine influences on key ECM components compared to MCI constructs. Nevertheless, MMPs and proinflammatory cytokines were affected by TNFα in different patterns when comparing MCI and ACT. This results in decreased ECM production, increased degradation and stimulation of inflammation. A major mechanism of increased sensitivity to external cytokine stimulation is the cell expansion process of the ACT technique. Consequently, the regeneration potential of cartilage fragments in MCI may be superior to the expanded chondrocytes of ACT in osteoarthritic clinical contexts. Further investigations are necessary for the translation of these findings into clinical practice.

## Data Availability

Data are contained within the article and Supplementary Materials.

## Supplementary Materials

The following supporting information can be downloaded at: https://www.mdpi.com/article/10.3390/cells13060546/s1, Figure S1: Negative controls for immunohistochemical staining of chondrocyte spheroid constructs and cartilage fragments for collagen 2 and 1.
