# Supplementary figures and images for: Comparison of Minced Cartilage Implantation with Autologous Chondrocyte Transplantation in an In Vitro Inflammation Model
Source: Cells. 2024 Mar 20;13(6):546. doi: 10.3390/cells13060546 (PMC10969176; doi:10.3390/cells13060546)

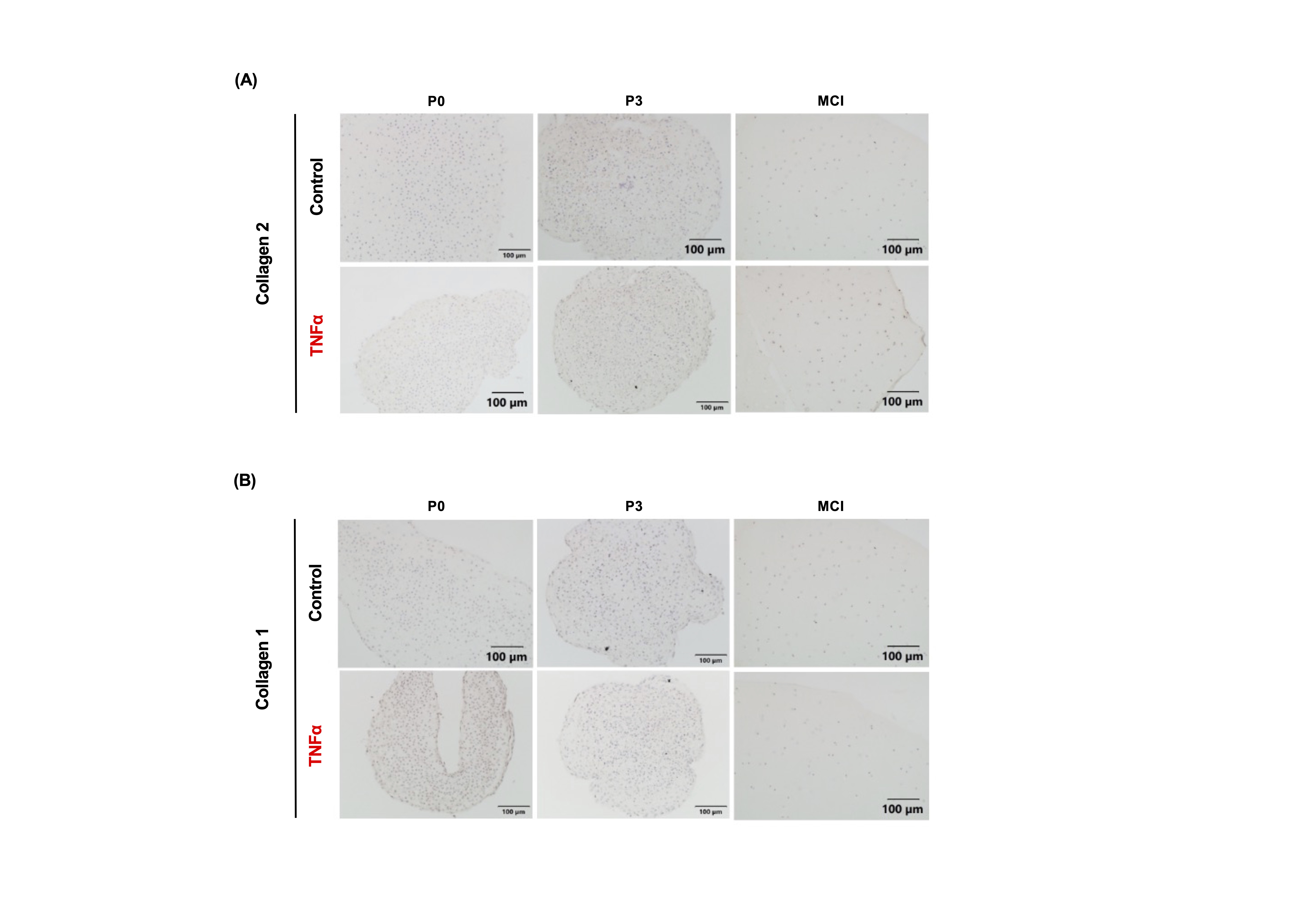

Supplement: Supplementary file 1 [file cells-13-00546-s001.zip › cells-2886327-supplementary.jpg]
